# Analysis of Neurology Residency Match Outcomes Using the Texas Seeking Transparency in Application to Residency (STAR) Database

**DOI:** 10.7759/cureus.96708

**Published:** 2025-11-12

**Authors:** Adam Y Ali, Samuel Salib, Niloufar S Tehrani, Gurkiranjeet Gakhal, Ryan Chowdhury, Ubaid Ansari, Layla Ali, Maaz Asim, Angela Mihalic, Michael S Wong

**Affiliations:** 1 Medicine, California Northstate University College of Medicine, Elk Grove, USA; 2 Otolaryngology - Head and Neck Surgery, California Northstate University College of Medicine, Elk Grove, USA; 3 Neurology, California Northstate University College of Medicine, Elk Grove, USA; 4 Orthopedic Surgery, California Northstate University College of Medicine, Elk Grove, USA; 5 Student Affairs, University of Texas Southwestern Medical Center, Dallas, USA; 6 Surgery/Plastic Surgery, California Northstate University College of Medicine, Elk Grove, USA

**Keywords:** clerkship honors, neurology residency, program signaling, residency application trends, texas star database, usmle step 1 pass-fail

## Abstract

Background

Historically, United States Medical Licensing Examination (USMLE) Step 1 scores played a crucial role in residency selection. However, recent trends have shifted toward a more holistic review process that emphasizes factors such as extracurricular activities and program location in addition to exam performance.

Objective

This study aims to identify predictors of successful matches into US-based neurology residency programs following the transition of USMLE Step 1 to pass/fail scoring.

Design/methods

This retrospective study analyzed neurology residency applicant data from the Texas Seeking Transparency in Application to Residency (STAR) database from 2019 to 2024. Following the IRB exemption, variables such as research output, leadership roles, Step 2 scores, clerkship honors, and program signaling were evaluated for their association with successful matches. ANOVA and t-tests were used to determine statistically significant predictors of match success.

Results

Among 1,017 matched neurology applicants, the mean number of abstracts presented increased from 2018 (4.65) to 2024 (5.63, *p* < 0.05). Volunteer experiences decreased significantly from 6.17 to 4.11 (*p* < 0.01). Clerkship honors predicted match success (*p* = 0.02) and peaked in 2024 (mean = 3.51). Among 2024 applicants who signaled a program, 47.1% matched to a signaled program. No significant changes were observed in research publications, leadership roles, Step 2 scores, or demographic factors.

Conclusions

Following the transition to pass/fail Step 1 scoring, research productivity and extracurricular activities remained important, while clerkship honors emerged as the strongest predictor of match success.

## Introduction

Over the past decade, the residency application process for neurology has undergone significant evolution, shaped by shifts in assessment criteria and broader systemic changes. One of the most transformative adjustments was the transition of the United States Medical Licensing Examination (USMLE) Step 1 to a pass/fail scoring system in 2022 [1]. Historically, Step 1 served as a key metric for stratifying applicants, offering program directors a standardized means of comparing academic performance across institutions. Its removal as a scored assessment has compelled both applicants and program directors to reassess what constitutes a competitive application.

As the weight previously placed on Step 1 has diminished, other elements such as research productivity, Step 2 clinical knowledge (CK) scores, clerkship honors, leadership roles, volunteer experiences, and program signaling have grown in perceived importance [1]. Simultaneously, the COVID-19 pandemic introduced new constraints, including virtual interviews, that have reshaped how programs evaluate applicants’ interpersonal skills and institutional fit [2,3]. These shifts raise pressing questions: Which metrics now best predict success in matching into neurology residency? How have applicant strategies adapted in response to these changes?

Existing literature offers limited insight into how these evolving criteria influence match outcomes, particularly in neurology. Many prior studies focus on aggregate trends across specialties or are outdated, given the rapid pace of recent reforms. Moreover, few studies account for the specific impact of structural barriers faced by applicants from underrepresented backgrounds, including social biases and differential access to mentorship and research opportunities [3,4]. As diversity, equity, and inclusion become focal points in medical education, understanding how these changes affect all demographics is crucial.

To address this gap, we analyzed self-reported data from the Texas Seeking Transparency in Application to Residency (STAR) survey, spanning the 2022-2024 match cycles. Our study aims to (1) compare the characteristics of matched versus unmatched applicants to neurology residency programs in the post-Step 1 pass/fail era and (2) identify which variables, specifically, research involvement, Step 2 CK scores, clerkship honors, leadership experiences, and volunteer work, serve as significant predictors of match success. By delineating these trends, we provide applicants, advisors, and program directors with evidence-based insights into the evolving definition of a competitive neurology applicant.

## Materials and methods

This retrospective study was conducted to analyze neurology residency match outcomes using data from the Texas STAR database. The study included all residency applicants from medical schools participating in the Texas STAR program who applied to neurology residency programs between 2019 and 2023. IRB exemption was obtained, as the study utilized de-identified data and posed minimal risk to participants.

Data sources

The Texas STAR database is a voluntary, national, multi-institutional program designed to provide transparency in the residency application process. Data included self-reported residency application metrics such as the USMLE scores, clerkship grades, number of research experiences, abstracts, posters and presentations, peer-reviewed publications, volunteer experiences, leadership positions, programs applied to, interview invitations, and match outcomes. Neurology applicants were identified from the overall dataset using the specialty of interest reported in the database.

Study population

The study included all neurology residency applicants who submitted surveys to the Texas STAR database between 2019 and 2024. In 2023, the survey received a 34% response rate, and in 2024, it received a 26% response rate. Applicants were classified as either matched (Y) or unmatched (N) based on their reported residency match outcomes. A total of 1,017 neurology residency applicants were included in the final analysis.

Data collection

Following IRB approval, a proposal outlining the study aims, methodologies, and expected outcomes was submitted, with contributions from a study advisor. A PubMed search using the keywords “neurology” + “residency” for publications from 2000 to 2024 was performed to contextualize our findings.

The Texas STAR database is a self-reported questionnaire completed by applicants. Texas STAR personnel were responsible for data extraction and provided the necessary datasets for analysis. The data was reviewed to ensure completeness and accuracy prior to statistical analysis. De-identified information was used to maintain confidentiality in accordance with data-sharing agreements. Inclusion criteria included all Texas STAR participants who applied to a neurology residency position between 2019 and 2024 with complete match outcome data (matched or unmatched). Exclusion criteria included all respondents with incomplete outcome fields or applicants listing multiple specialties without specifying neurology as their primary interest. Criteria measured included abstracts/posters/presentations, publications, research experiences, leadership positions, volunteer experiences, Step 2 scores, and clerkship honors. 

Risk of bias assessment

The primary risk of bias includes those associated with retrospective studies on self-reported survey databases, including self-report bias, as applicants may over- or under-report their respective achievements, and selection bias, since response rates varied. To minimize this, we only included responses with complete outcome and experience data along with descriptive analyses.

Statistical analysis

Statistical analysis was conducted using IBM SPSS Statistics for Windows, Version 28.0 (Released 2021; IBM Corp., Armonk, NY, USA). Descriptive statistics, including mean, standard deviation, and frequencies, were calculated to summarize the demographic characteristics and application profiles of neurology applicants. ANOVA was used to compare continuous variables such as USMLE scores across different match years, while T-tests were conducted to evaluate significant differences between matched and unmatched groups. A p-value of less than 0.05 was considered statistically significant for all analyses.

Further exploratory analysis was performed to assess trends in match rates over time and to identify factors associated with successful match outcomes. These factors included, but were not limited to, applicant qualifications (e.g., USMLE Step 1 and Step 2 scores, number of interview invites, and research productivity) and the number of applications submitted.

An important development in 2024 was the adjustment to the Electronic Residency Application Service (ERAS) application’s “Experience” section, limiting applicants to a total of 10 experiences across volunteer, research, work, and extracurricular activities. To account for these changes, results are compared across two distinct periods: 2018-2023 (pre-Step 1 policy change) and 2023-2024 (post-ERAS limit on experiences). In 2024, the Texas STAR survey also asked respondents to state how many experiences they reported to the National Resident Matching Program (NRMP) in order to minimize the impact on ERAS experience limitations.

Ethical considerations

All data were de-identified prior to analysis, and an IRB exemption ensured compliance with ethical guidelines for retrospective studies. The results of the analysis were used solely for research purposes to better understand the residency application process and to identify potential areas for improvement in the neurology match process.

This methodology ensures a comprehensive and rigorous approach to analyzing the residency match outcomes of neurology applicants in the Texas STAR database from 2018 to 2024.

## Results

Research

For matched neurology residency applicants, the only statistically significant change in research output was observed in the mean number of abstracts, posters, and presentations (2023: mean = 4.94, SD = 4.12; 2024: mean = 5.63, 95% CI 5.05-6.22, p < 0.05). However, the values in 2024 reflect the average of the mean value of abstract posters and presentations prior to 2023 (2018-2022: mean = 5.63, SD = 3.89).

The mean number of publications decreased from 2023 (mean = 3.09, SD = 3.35) to 2024 (mean = 2.75, 95% CI 2.26, 3.24), though this change is not statistically significant (p = 0.2508). Research experiences followed a similar trend, decreasing from 2023 (mean = 4.16, 95% CI 3.77, 4.55) to 2024 (mean = 3.94, 95% CI 3.50, 4.38, p = 0.4439). These results are presented in Table 1.

**Table 1:** Curricular and non-curricular factors in neurology match

Year	2018		2019		2020		2021		2022		2023		2024	
Metric	Mean	SD	Mean	SD	Mean	SD	Mean	SD	Mean	SD	Mean	SD	Mean	SD
Abstracts/posters/presentations	5.63	3.89	4.44	3.5	4.8	3.87	5.15	3.91	5.19	3.99	3.79	4.94	4.12	5.63
Publications	2.75	3.25	1.91	2.36	2.62	2.97	2.89	3.1	2.88	3.26	2.9	3.09	3.35	2.75
Research experiences	3.94	2.91	4.17	2.45	4.11	2.58	4.36	2.52	4.34	2.69	2.48	4.16	2.62	3.94
Leadership positions	3.25	1.86	3.67	2.63	3.4	2.53	4.01	2.77	3.79	2.32	2.07	3.86	2.67	3.24
Volunteer experiences	4.12	2.67	6.41	3.17	6.72	3.34	6.94	3.25	6.43	3.1	2.92	7.24	3.23	4.12
Step 2 scores	218.25	4.79	221	7.38	220.93	5.94	220.53	6.56	221.55	6.11	5.01	225	3.16	218.25
Clerkship honors	3.51	2.32	3.43	2.54	3.14	2.39	2.61	2.32	3.16	2.36	2.21	2.99	2.51	3.51

**Figure 1:**
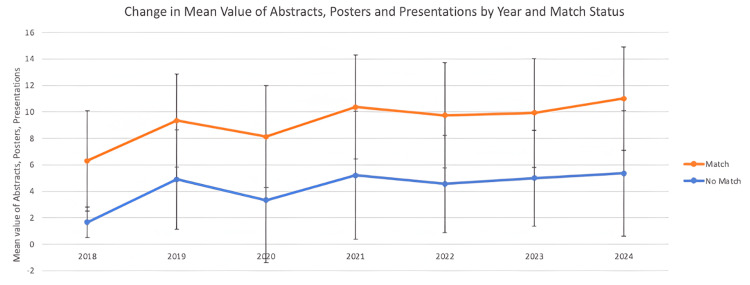
Abstracts, posters, and presentations by year and match status

Figure 1 illustrates the trends in abstracts, posters, and presentations among neurology residency applicants, comparing those who matched successfully with those who did not. From this figure, we observe that research activity has consistently increased among successful applicants over time.

Extracurricular aspects

**Figure 2:**
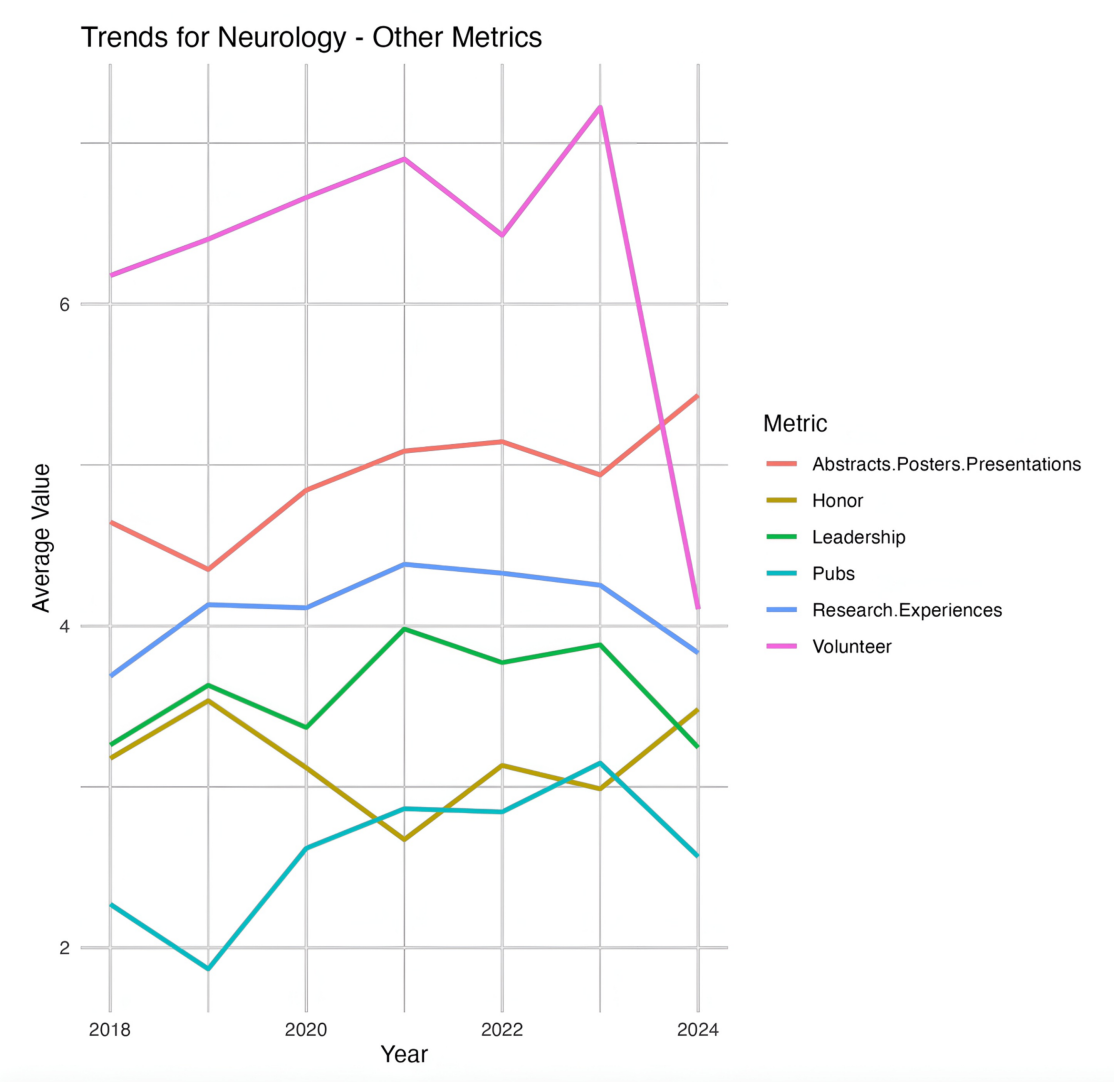
Trends for neurology: other metrics

Volunteer experiences were the only statistically significant metric to decrease significantly between 2023 and 2024 (p < 0.01). It is worth noting that the ERAS changed its application, so there is a limit of 10 experiences. Leadership positions also showed a mild decrease between 2023 (mean = 3.86, SD = 2.67, 95% CI 3.46, 4.27) and 2024 (mean = 3.24, SD = 1.86, 95% CI 2.97, 3.53). This overall change was not statistically significant (p = 0.9628). Table 1 depicts all these changes, and Figure 2 illustrates how leadership positions have changed from 2018 to 2024.

Curricular metrics

The only statistically significant predictor of match success was observed with clerkship honors (p < 0.05). Clerkship honors increased from 2018 (mean = 3.20, SD = 2.21, 95% CI 2.77, 3.64) to 2024 (mean = 3.51, SD = 2.32, 95% CI 3.15, 3.87) (Table 1).

**Figure 3:**
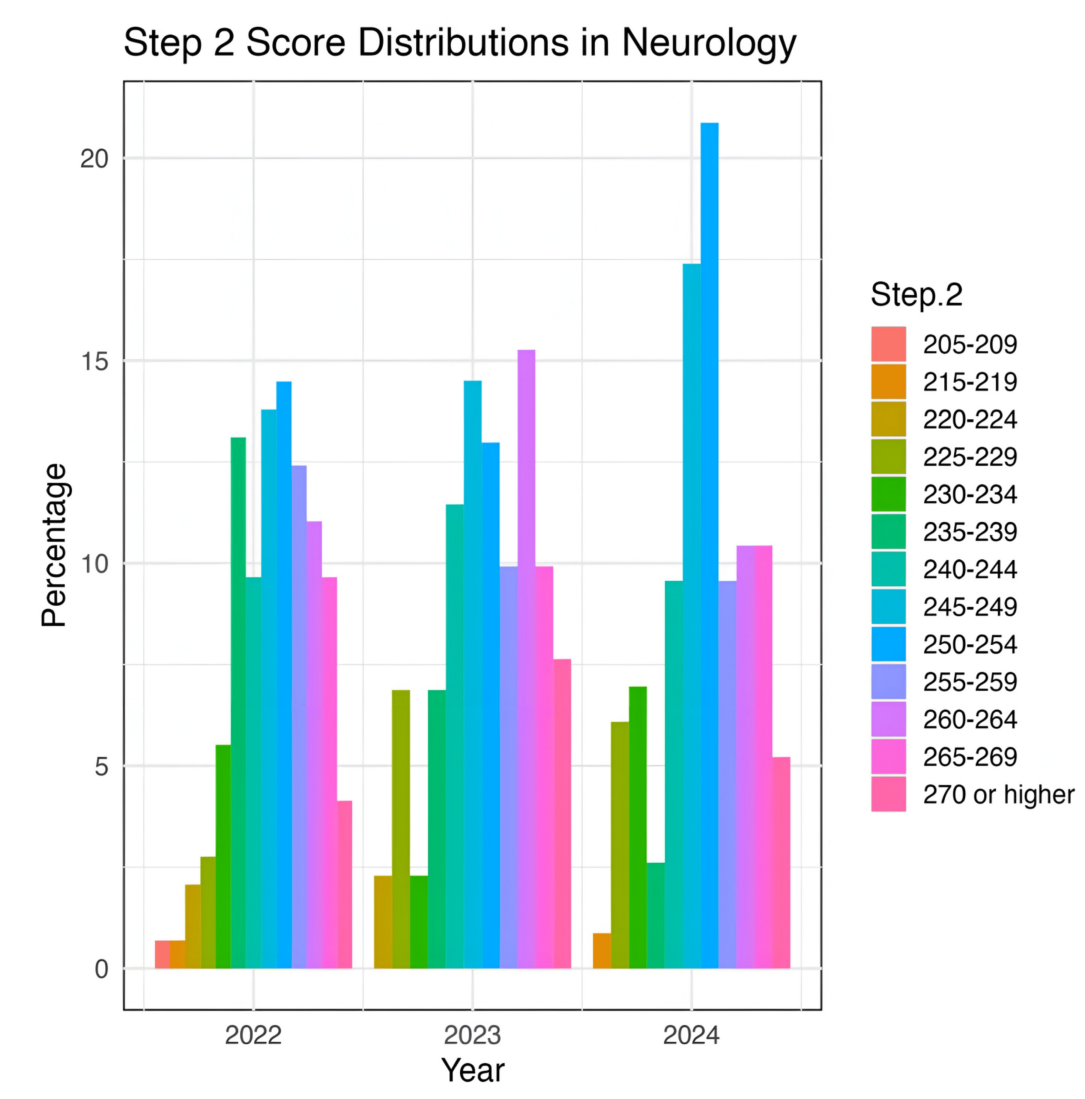
Distribution of Step 2 scores in neurology

Step 2 scores saw a decrease from their average in 2023 (mean = 225, SD = 3.16) to 2024 (mean = 218.25, SD = 4.79). This change was not statistically significant (p = 0.4547). Step 2 score distributions for the years 2022-2024 are depicted in Figure 3.

Program signaling

Regarding program signaling, it appears to be an increasingly predictive factor in match outcomes. In 2023, 45.3% of applicants matched with a signaled program, and this figure rose to 47.1% in 2024.

Application cycle

In 2018, neurology applicants applied to an average of 28.74 programs (SD = 18.46, 95% CI 25.09, 32.40). By 2023, this mean had increased to 35.37 (SD = 18.09, 95% CI 32.66, 38.07), before slightly decreasing to 34.55 in 2024 (SD = 18.41, 95% CI 31.82, 37.27). This overall change from 2018 to 2024 was statistically significant (p < 0.05).

The average number of interview offers received by applicants decreased significantly (p < 0.05) from 17.41 in 2018 (SD = 8.88, 95% CI 15.65, 19.17) to 14.85 in 2024 (SD = 5.86, 95% CI 13.98, 15.72). This falls in line with the increase in program signaling in 2023 and 2024.

The number of interviews attended by applicants increased from an average of 13.01 in 2018 (SD = 4.45, 95% CI 12.13, 13.89) to 14.33 in 2023 (SD = 5.25, 95% CI 13.54, 15.11), with a slight decrease to 14.18 in 2024 (SD = 4.40, 95% CI 13.53, 14.84). This change over time was also statistically significant (p = 0.0374).

Demographics

**Figure 4:**
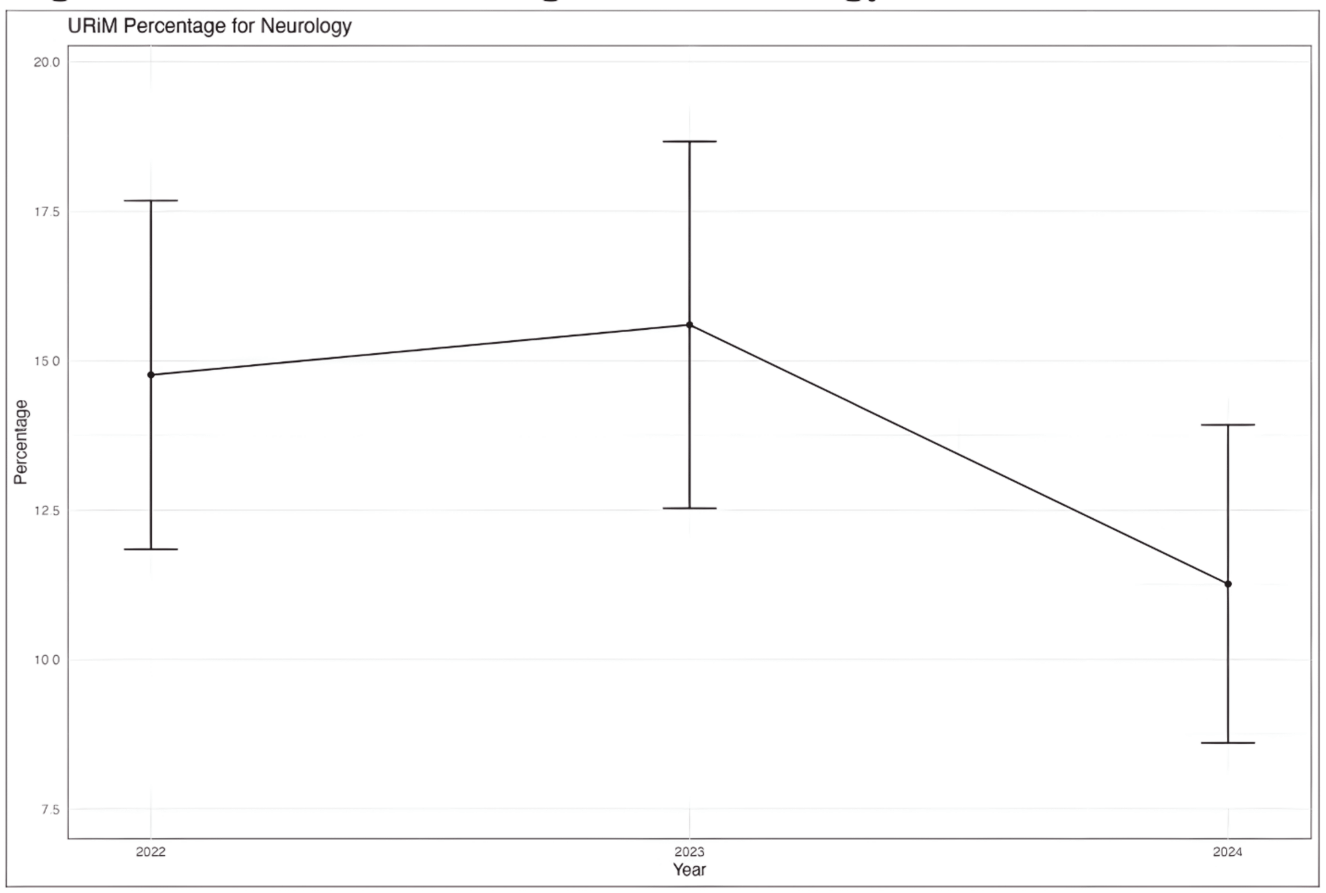
Percentage of underrepresented minorities (URiM) in neurology

The proportion of underrepresented minorities (URiM) in neurology residency applications declined from 16% in 2023 to 10% in 2024, though this value is not statistically significant (Figure 4). Female applicants increased slightly, from 54.2% in 2023 to 59.1% in 2024.

## Discussion

In 2022, the USMLE Step 1 exam transitioned from a scored to a pass-fail system, affecting applicants in residency programs in the class of 2024. This change was implemented to alleviate exam-related burden on medical students. However, this shift may have influenced the priorities of both applicants and program directors, leading to changes in the content and strengths of applications compared to pre-policy years.

Research

Certain research activities have become increasingly important among successful neurology applicants. For instance, only research activities such as abstracts, posters, and presentations rose significantly (from 4.65 in 2018 to 5.63 in 2024). And while these types of research activities have increased, the number of manuscript publications and overall research experiences have not changed significantly. Therefore, abstracts, posters, and presentations remain a crucial component of research output for successful applicants.

This change in research output for successful neurology residency applicants could reflect either a greater pattern among all program directors to increase research productivity in their own departments or a desire for students to make themselves more attractive applicants to residency program directors. One study at New York University found that, among orthopedic residents, the number of articles published before residency was positively associated with the number of articles published during residency [5].

Additionally, program directors have reported that meaningful research participation can demonstrate various positive attributes among residents, such as intellectual curiosity, critical thinking skills, and self-directed learning skills [5]. A possible explanation for this is Kolb’s Learning Theory, based on experiential learning, which highlights the importance of personal experience in the learning process. According to Kolb, individuals learn through a cyclical process involving four stages: concrete experience, reflective observation, abstract conceptualization, and active experimentation [6]. Each stage reflects a different type of learning style, which explains the effectiveness of this model to describe the learning process of future residents.

A notable factor in research participation is that such skills are only likely to develop if research is well-mentored and adequately resourced, hence the term meaningful [7]. Therefore, since the 2022 change of USMLE Step 1 to pass-fail, 41% of program directors have reported that meaningful research participation will be more important in offering interviews. It is worth noting that the type of training program will likely play a role in the importance of research output, since 68.2% of academic medical centers or university-affiliated settings claimed that they had a primary goal of training clinicians and scientists, compared to 43.9% of community-based settings and 71.4% of military or federal settings [5].

On the other hand, the gap in research output between matched and unmatched applicants has also narrowed over time, with matched applicants reporting 5.63 abstracts and posters compared to 5.36 among unmatched candidates in 2024. This could reflect a greater emphasis on research output among all residency applicants, which encourages students to engage in research regardless of their holistic qualifications such as Step 2 scores or other metrics.

Leadership and volunteering

The data show general increases from 2018 to 2022/2023 in leadership and volunteer experiences, followed by a drop, possibly influenced by recent changes in application constraints. Volunteer activities increased from 6.17 in 2018 to 7.24 in 2023, but dropped significantly in 2024 to 4.12 experiences, likely reflecting the ERAS limitation on reported experiences. Leadership experiences also saw a minor rise from 3.26 to 3.86 between 2018 and 2023, with a decrease to 3.24 in 2024, though this change was not statistically significant.

The limitation of 10 experiences in the ERAS application forces applicants to be more selective in how they present their experiences and likely impacts how applicants represent their extracurricular activities, with many possibly favoring research to meet program expectations [8]. This prioritization may compress and reshape extracurricular profiles, making it challenging to compare applicant profiles consistently across years. Future analysis of these shifts will be essential for understanding long-term trends in neurology residency applications.

Changes in the application process

The onset of the COVID-19 pandemic in 2020-2021 introduced virtual interviews, which have since become a fixture in the residency application process. The American Academy of Neurology has advised all programs to continue to conduct virtual interviews throughout the 2023-2024 application cycle [9]. The consistency in match success rates from pre-pandemic years to 2024 indicates the resilience of this virtual model, suggesting that the format change has not negatively impacted outcomes [9]. Specialized study modules (SSMs) have also demonstrated effectiveness in enhancing student engagement and outcomes, which parallels this finding from virtual assessment environments where match success remained stable post-pandemic [10].

Program signaling has also gained prominence, allowing applicants to indicate interest in specific programs. Residency applicants to adult neurology are allowed to signal up to eight programs, and applicants to child neurology can signal up to three [11]. Signaling appears to align applicants’ interests with program preferences, potentially reducing geographic biases and supporting a selective interview process [12]. As virtual interviews simplify attendance logistics, applicants can attend more interviews, enhancing match chances while programs concentrate on candidates with demonstrated interest [11]. Before, since the online format of interviews has made it easier for applicants to attend the interviews they receive, signaling emerged out of necessity to help program directors better understand the seriousness of interviewees at their programs [7]. Now, this alignment between applicant preferences and interview offers may lead to fewer interviews overall but a higher attendance rate [7]. In fact, the AAMC properly predicted that the signal-to-interview conversion rates would be high across the board for the 2024 ERAS cycle [7]. Together, signaling and virtual interviews are reshaping neurology residency applications, influencing applicant and program behaviors and setting a potential standard for future application cycles.

Step 2 exam scores

With Step 1 now pass-fail, Step 2 scores may assume greater weight in applications. Our analysis shows a stable trend in Step 2 scores from 2018 to 2024, with averages of 218.42 to 225, and no significant change in recent years. Step 2 scores are shown to be the only variable that has a meaningful correlation with Step 1 scores [13]. Additionally, no other objective metrics, such as research output, were correlated with Step 1 scores, which further justifies how it might be seen as the Step 1 replacement [13]. However, the difference between matched and unmatched step 2 scores has decreased. These differences in the Step 2 score distributions would indicate a different cohort of applicants for interview, perhaps with more holistic qualifications, which further indicates the need for other methods to screen candidates [13].

Demographics

In terms of demographics, there were no statistically significant shifts in the proportions of gender, URiMs, or first-generation applicants among neurology candidates. Though non-significant, trends in the data suggest possible declines in the proportions of male, URiM, and first-generation applicants. One study at the University of Ohio utilized a multifaceted strategy to increase the percentage of URiM residency applicants who matched into their pediatric residency programs, such as child neurology. The percentage of URiM who entered their pediatric residency programs changed from 15% in 2018 to 22% in 2021 [14]. However, given the overall lack of change in demographic trends among neurology residency applicants, external factors, such as changes in diversity policies and economic pressures, may be influencing the stability of these metrics. Other hindrances to increased URiM involvement could be pipeline concerns or decreased recruitment during the pandemic [14]. Additionally, the “minority tax,” also referred to as the additional responsibilities minority trainees take in participating in diversity initiatives, may hinder professional advancement and disrupt training [14]. Finally, the 2023 Supreme Court ruling on Affirmative Action, for example, could impact URiM and first-generation applicant rates in residency programs. However, these points remain speculative, and further research is needed to determine the impact of such factors.

Implications for practice

These findings highlight several evolving considerations for applicants, program directors, and medical educators. For applicants, a balanced approach that emphasizes research in the form of abstracts, posters, and presentations, while strategically selecting a limited number of meaningful experiences under the ERAS 10-entry cap, may offer a competitive edge. As research output becomes more important and Step 1 is no longer a differentiator, applicants should consider engaging in mentored projects that demonstrate initiative, critical thinking, and sustained involvement.

For program directors, the growing role of signaling offers a tool to better identify applicants who have a genuine interest in specific programs. This may streamline interview selection, reduce regional bias, and improve the quality of interview pools. Additionally, as Step 2 scores take on greater importance and other metrics like leadership or volunteerism become harder to assess due to reporting limitations, programs may need to adopt more holistic and contextualized approaches to reviewing applicants.

For medical educators and institutions, these findings point to the need for early mentorship and clearer advising around the evolving residency application landscape. Supporting students in identifying high-yield research and leadership opportunities, preparing for Step 2 early, and navigating the new ERAS structure will be essential. As application behaviors continue to shift, further research will be critical in evaluating how these changes affect applicant equity, program selection strategies, and overall match outcomes.

Ethical considerations and limitations

The Texas STAR database includes data only from applicants who voluntarily completed the survey, introducing a risk of sampling bias. Furthermore, although most questions are mandatory, select demographic variables, including race/ethnicity, food and housing insecurity, and first-generation status, are optional, introducing potential voluntary response bias. In addition, because the survey is self-reported and retrospective, it is subject to both recall and selection biases. These limitations should be acknowledged when interpreting trends or drawing conclusions from the dataset.

From an ethical standpoint, transparency about data sources and methodology is essential for building trust and ensuring responsible use of findings. While the Texas STAR dataset is a powerful and widely used tool for understanding residency trends, its reliance on self-reported information from a non-random sample limits its generalizability. As such, care must be taken to avoid overgeneralizing trends or using the data to make overly deterministic judgments about applicant competitiveness. Addressing these ethical considerations more explicitly in both research and advising contexts would improve the clarity, credibility, and utility of future analyses.

## Conclusions

In conclusion, from 2018 to 2024, neurology residency applicants in the USA have shifted their application focus, reflecting changes in USMLE Step 1 scoring. Following the transition of pass-fail Step 1, research output and extracurriculars remained important, while clerkship honors emerged as the strongest predictor of match success. While the demographic composition remains stable, applicants appear increasingly focused on research publications, possibly at the expense of leadership and volunteering. If this trend continues, other areas of the application could be impacted. Additional data on matriculated neurology residents would provide a clearer understanding of evolving applicant expectations in the field.
